# Coping with Persistent Pain, Effectiveness Research into Self-management (COPERS): statistical analysis plan for a randomised controlled trial

**DOI:** 10.1186/1745-6215-15-59

**Published:** 2014-02-15

**Authors:** Brennan C Kahan, Karla Diaz-Ordaz, Kate Homer, Dawn Carnes, Martin Underwood, Stephanie JC Taylor, Stephen A Bremner, Sandra Eldridge

**Affiliations:** 1 Pragmatic Clinical Trials Unit, Queen Mary University of London, 58 Turner St, London E1 2AB, UK; 2 Department of Health Services Research and Policy, London School of Hygiene and Tropical Medicine, 15-17 Tavistock Place, London WC1H 9SH, UK; 3 Translational Research Unit, Centre for Primary Care and Public Health, Blizard Institute, Barts and The London School of Medicine and Dentistry, Queen Mary University of London 58 Turner St, London E1 2AB, UK; 4 Warwick Clinical Trials Unit, Warwick Medical School, Gibbett Hill Road, Coventry CV4 7AL, England

**Keywords:** Statistical analysis plan, Randomised controlled trial, Self-management, Chronic musculoskeletal pain, Complex intervention

## Abstract

**Background:**

The Coping with Persistent Pain, Effectiveness Research into Self-management (COPERS) trial assessed whether a group-based self-management course is effective in reducing pain-related disability in participants with chronic musculoskeletal pain. This article describes the statistical analysis plan for the COPERS trial.

**Methods and design:**

COPERS was a pragmatic, multicentre, unmasked, parallel group, randomised controlled trial. This article describes (a) the overall analysis principles (including which participants will be included in each analysis, how results will be presented, which covariates will be adjusted for, and how we will account for clustering in the intervention group); (b) the primary and secondary outcomes, and how each outcome will be analysed; (c) sensitivity analyses; (d) subgroup analyses; and (e) adherence-adjusted analyses.

**Trial registration:**

ISRCTN24426731

## Update

### Background

**Figure 1:**
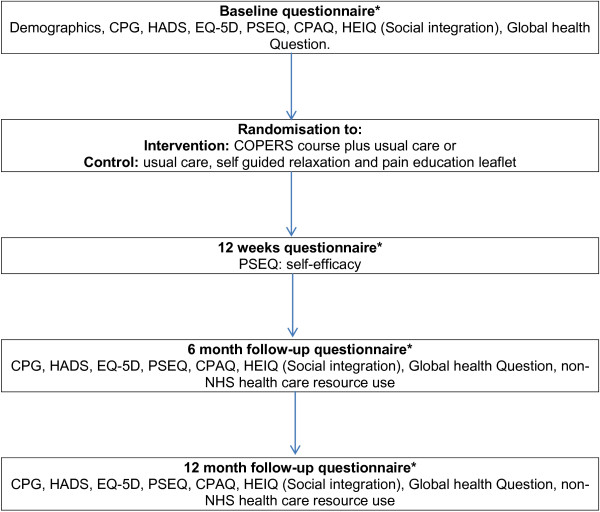
**Schedule of assessment.** CPG, Chronic Pain Grade (Von Korff [3]); HADS, Hospital Anxiety and Depression Scale (Zigmond and Snaith [5]); EQ-5D, Quality of life (EuroQol.org); PSEQ, Pain Self-Efficacy Questionnaire (Nicholas 1989, [4]); CPAQ, Chronic Pain Acceptance Questionnaire (McCracken [6]); HEIQ, Social integration [7]; Census global health question (ons.gov.uk/census/2011-census).

COPERS (Coping with Persistent Pain, Effectiveness Research into Self-management) was a pragmatic, multicentre, unmasked, parallel group, randomised controlled trial [1]. The protocol for the COPERS trial has been published previously [1] and gives details on the rational for the trial, the intervention and control groups, the inclusion/exclusion criteria, and the sample size calculation. Briefly, the main aim of the COPERS trial was to assess whether a group-based learning course was effective in reducing pain-related disability in participants with chronic musculoskeletal pain [2]. The intervention was a 3-day training course. Course content is shown in Table 1, and the schedule of assessments is shown in Figure 1. The intervention was compared with usual care plus a relaxation CD and pain education leaflet. In this article, we describe the statistical analysis plan, which outlines how data analysis for the COPERS trial will be performed.

The statistical analysis plan was finalised and approved on 3 October 2013. All trial investigators were blinded to patient outcomes broken down by treatment group until after the analysis plan was signed off and the database was locked. Participant recruitment began in August 2011 and finished in July 2012 (participant follow-up was completed in August 2013). The trial database was locked after the statistical analysis plan was approved. Data analysis did not begin until after the analysis plan was approved, and after the database was locked.

**Table 1:** The Coping with Persistent Pain, Effectiveness Research into Self-management (COPERS) course content overview

**Day (10.00 am to 2.45 pm)**	**Modules**
**Day 1**	Introduction and understanding pain and acceptance
Living and dealing with pain	
	Lunch
	Activity: Art
	Mind, mood and pain
	Movement and posture, and Relaxation
**Day 2**	Dealing with unhelpful, negative thoughts and barriers to change
Doing something about your life with pain	
	Lunch
	Activity: Hand massage
	Making pain more manageable
	Movement and Relaxation
**Day 3**	Communication skills
Communication and relationships	Lunch
	Activity: Volunteering
	Movement and Relaxation
**Day 4**	The future
Follow-up (2 weeks later, 10.00 am to 12.00)	

The original sample size calculation called for 685 participants (391 in the intervention group and 294 in the control group, to be randomised in a 4:3 ratio). However, we required a sufficient number of participants in each group-based learning course to make the intervention viable; therefore, we continued recruitment until there was an adequate number of participants for each course. This led to 703 participants (403 intervention, 300 control).

Ethics approval was granted by Cambridgeshire Ethics 11/EE/04. All patients provided written informed consent prior to randomisation.

### Outcomes

#### Primary outcome

The primary outcome was the pain-related disability score (which is a subscale of the Chronic Pain Grade questionnaire (CPG disability) [3]) at 12 months post-randomisation.

The three questions in this subscale assess the extent to which the participant’s pain has, in the previous 6 months: (i) interfered with their ability to perform their daily activities; (ii) changed their ability to take part in recreational, social, and family activities; and (iii) changed their ability to work. Each of the three questions is rated on a scale of 0 to 10, with 0 reflecting no change or interference, and 10 reflecting extreme change or interference.

The primary outcome is the mean of these three questions, multiplied by 10; that is, if X1, *X*2, and X3 represent the three questions, and Y represents the primary outcome, then:

$$Y=10*\left(X1+X2+X3\right)/3$$

The primary outcome is therefore recorded on a scale from 0 to 100, with higher scores reflecting larger interference or change in the participant’s ability to perform daily activities, work, or take part in recreational, social, and family activities.

#### Secondary outcomes

The secondary outcomes are:

1. CPG disability at 6 months post-randomisation [3]

2. CPG pain intensity score at 6 and 12 months post-randomisation [3]

3. Pain Self-Efficacy Questionnaire (PSEQ) score at 6 and 12 months post-randomisation [4]

4. Hospital Anxiety and Depression Scale (HADS) Anxiety score at 6 and 12 months post-randomisation [5]

5. Hospital Anxiety and Depression Scale (HADS) Depression score at 6 and 12 months post-randomisation [5]

6. Chronic Pain Acceptance Questionnaire (CPAQ) score at 6 and 12 months post-randomisation [6]

7. Health Education Impact Questionnaire (HEIQ) Social integration score at 6 and 12 months post-randomisation [7]

8. EQ-5D at 6 and 12 months post-randomisation [8]

9. Census global health question at 6 and 12 months post-randomisation [9]

10. Total Defined Daily Doses (Total DDD) of psychotropic drugs consumed up to 12 months post-randomisation

11. Total DDD of analgesics (including all opioids and other central nervous system drugs) consumed for pain up to 12 months post-randomisation

12. Total DDD of weak opioids consumed up to 12 months post-randomisation

13. Total DDD of strong opioids consumed up to 12 months post-randomisation

14. Proportion of participants using weak opioids at 12 months post-randomisation (defined as having received a prescription for a weak opioid up to twelve weeks before the 12-month follow-up date)

15. Proportion of participants using strong opioids at 12 months post-randomisation (defined as having received a prescription for a strong opioid up to twelve weeks before the 12-month follow-up date)

A guide to how outcomes are derived is available in Additional file 1.

### Analysis principles

#### General analysis principles

The main analysis for each outcome will use intention-to-treat (ITT) principles, meaning that all participants with a recorded outcome will be included in the analysis, and will be analysed according to the treatment group to which they were randomised [10]. More information on which participants will be included in each analysis is available in later sections. All *P* values will be two sided, and the significance level is set at 5%.

Analyses for all outcomes will be presented as:

1. The number of participants included in the analysis, by treatment group;

2. A summary measure of the outcome, by treatment group (for example, mean (SD) for continuous outcomes, number (%) for binary outcomes). Only participants with a completely recorded outcome will be used to calculate the summary measure (for example, participants who complete only one of three components of the CPG disability score will not be included in the calculation of the summary measure, although they will be included in estimated the treatment through the use of multiple imputation - see later sections);

3. A treatment effect, with a 95% confidence interval;

4. A two-sided *P* value.

All analyses will account for clustering by course in the intervention group. This is because when the intervention is delivered in clusters (for example, in a group setting, or when the same therapist treats multiple patients) it is necessary to account for the clustering in the analysis to avoid increasing the type I error rate [11-13]. Participants in the control group (who do not attend courses), will act as their own cluster (that is, each participant in the control group will belong to a 'course’ where they are the only member).

Site of recruitment (London or Midlands) [14-17], age, gender, and the HADS depression score at baseline will be included as covariates in each analysis [17-20]. Additionally, for continuous outcomes (CPG disability, CPG pain intensity, PSEQ, HADS Anxiety, HADS Depression, CPAQ, HEIQ, and EQ-5D), the outcome measured at baseline will be included in the analysis [19]. Site of recruitment will be adjusted for because it was used as a stratification factor in the randomisation process, and therefore, it is necessary to include it in the analysis to maintain correct type I error rates [11,14-16]. The other covariates will be included in the analysis because adjustment for prognostic factors can substantially increase power [17-21].

Continuous covariates (age, HADS depression score, outcome measured at baseline) will be analysed using a linear relationship with the outcome, as categorising continuous covariates has been shown to lead to poor performance in other scenarios [22]. We made the decision to assume a linear relationship prior to examining the observed association between covariate and outcome in the trial data, as previous research has shown that choosing the analysis method based on study data can lead to biased treatment effect estimates and incorrect type I error rates in some scenarios [23-27].

### Analysis of primary outcome

The primary outcome (CPG disability at 12 months) will be analysed using a mixed-effects linear regression model, with a 'group-based learning course’ as a random effect. Restricted maximum likelihood (REML) will be used. The model will include site of recruitment, age, gender, HADS depression score, and CPG disability at baseline as covariates.

All participants who completed at least one of the three questions that form the CPG disability score at either 6 or 12 months will be included in the analysis. Participants who did not fill out any portion of the CPG disability score at both 6 or 12 months will be excluded from the analysis.

Multiple imputation (MI) [28] will be used to account for participants who have an observed outcome at 6 months, but are missing the outcome at 12 months, as well as for participants who completed some, but not all, of the questions on the CPG disability score at 12 months. A total of 20 imputations will be performed, and results will be combined using Rubin’s Rules [28]. Only participants included in the analysis will be included in the imputation model. The imputation model will include the three questions that form the CPG disability score at baseline, 6 months, and 12 months, as well as the site of recruitment, age, gender, the HADS depression score at baseline, and employment status (employed or in full-time education versus not employed or in full-time education) (14 variables in total). We chose to include these variables in the imputation model because they will be included in the analysis model (CPG disability score at baseline, 6 months, and 12 months, site of recruitment, age, gender, and HADS depression score at baseline) or because we felt they may be predictive of missingness (employed or in full-time education versus not employed or in full-time education). Imputation will be performed separately within each treatment group [29]. In the intervention group, multilevel imputation will be performed, with 'course’ included in the imputation model as a random effect [30,31].

Missing data in any of the covariates to be adjusted for in the analysis (site of recruitment, age, gender, HADS depression score, CPG disability and baseline) will be accounted for at the same time as missing data in the CPG disability score at 6 or 12 months, as these variables will be included in the same imputation model.

### Sensitivity analyses for primary outcome

#### Method of accounting for missing data

We will perform three sensitivity analyses for the primary outcome to assess the robustness of the results to other methods of account for missing data [32]. The first sensitivity analysis involves specifying a different imputation model than that used in the primary analysis, and the last two sensitivity analyses involve the re-analysis of the primary outcome using two approaches which are not based on MI.

The sensitivity analyses are:

1. We will determine which baseline covariates are associated with loss to follow-up and include them in the imputation model. The analysis model will be the same as that for the primary analysis, except for the inclusion of additional covariates (that is, those that we determined were associated with loss to follow-up) in the imputation model.

2. We will perform a complete case analysis, where all participants who did not complete all components of the CPG disability score at 12 months will be excluded from the analysis. The analysis model will be the same as that for the primary analysis, except missing baseline covariates will be replaced using mean imputation. We used mean imputation as it has been shown to give unbiased estimates and standard errors for the treatment effect in randomised trials, and will suffer only a small loss in efficiency when the amount of missing data is small [33].

3. We will analyse the three components that form the CPG disability score at 12 months, rather than the overall CPG disability score. This will be done by performing a multivariate analysis, where each of the three components from the 12-month score are included in the model as outcomes (that is, each participant will have three outcomes). A three-level mixed-effects model will be used, with random effects for 'course’ and for participant. Treatment-by-question interactions will be included, allowing the treatment effect to vary for each of the three components. An overall treatment effect for CPG disability at 12 months will be estimated using the *lincom* function in Stata (StataCorp, College Station, TX, USA) to combine the treatment estimates from the three separate components. As above, missing baseline covariates will be replaced using mean imputation.

#### Participants with no completed follow-ups

The primary analysis has assumed that the excluded participants (those not completing any questions on the CPG disability questionnaire at both 6 and 12 months) were missing-at-random (that is, they were missing based on the covariates included in the analysis model). To assess the robustness to departures from this assumption, the primary outcome will be assessed under a range of missing-not-at-random scenarios. This will be done using the formula

$$\Delta =\Delta_{\mathrm{primary}}+Y_{1}P_{1}-Y_{2}P_{2}$$

where ∆ is the treatment effect under the missing-not-at-random scenario, ∆_primary_ is the treatment effect from the primary analysis, Y_1_ and Y_2_ are the assumed mean responses for participants with missing data in treatment groups 1 and 2 respectively, and P_1_ and P_2_ are the proportion of participants who were excluded from the analysis in groups 1 and 2, respectively. The standard error for ∆ is assumed to be approximately equal to the standard error for ∆_primary._ Y_2_ will be varied between 10, 25, 50, 75, and 90, and for each value of Y_2_, Y_1_ will be set to Y_2_ - 10, Y_2_, and Y_2_ + 10. For example, for Y_2_ = 25, Y_1_ will vary between 15, 25, and 35.

#### Re-definition of primary endpoint

The primary outcome is a composite of three questions. The first question (Q1) assesses to what extent the participant’s pain has interfered with daily activities in the previous 6 months. This is assessed on a scale of 0 to 10, with higher scores indicating more interference. The last two questions assess to what extent the participant’s pain has changed their ability to (a) take part in recreational, social, and family activities (Q2); and (b) work (Q3). Both these questions are measured on a scale from 0 to 10, with higher scores indicating more extreme change.

For the last two questions, higher change scores are meant to represent a higher *negative* change. However, it is possible that some participants have misinterpreted this, and have recorded a high score to indicate a large positive change. We will therefore perform a sensitivity analysis by redefining the outcome for participants whose scores indicate they may have misinterpreted the intended direction of the questions relating to change.

For participants with a score of 2 or less for Q1 (indicating very little interference in daily activities) *and* a score of 8 or higher on either Q2 or Q3 (intending to indicate an extreme negative change in their ability to take part in social activities or to work), we will assume the participant has misinterpreted the intended direction of the scale for Q2 or Q3 (as it is inconsistent for the pain to have had very little interference in daily activities, and for there to have been an extreme negative change in the participant’s ability to take part in activities or work). We will therefore rescore Q2 or Q3 based on a reverse scale (that is, a score of 10 will be rescored as 0, 9 will be rescored as 1, and 8 will be rescored as 2). We will then re-analyse the outcome using the same method as for the main analysis.

### Subgroup analyses

Subgroup analyses will be performed for the primary outcome (CPG disability at 12 months). All subgroup analyses will be performed using the same analysis model as for the primary outcome, but will also include the subgroup of interest and a treatment-by-subgroup interaction. Interaction tests will be considered significant at the 5% level. No correction will be made for multiple tests.

The following subgroups will be assessed:

#### Non-pain

•Comorbidity (number of body systems affected): ≤3 versus >3 comorbidities (with chronic musculoskeletal pain counting as a comorbidity)

•Living arrangements: living alone versus living with others

•Baseline self-efficacy: PSEQ score 0 to 20 (not likely to be confident) versus 21 to 39 (more likely to be confident and to self manage) versus ≥40 (confident)

•Socioeconomic status (SES) (based on the Index of Multiple Deprivation 2010, calculated from participant postcodes via Geographical Information Systems (GIS): lower social class (less than observed median in data) versus higher social class (equal or greater than observed median in data)

#### Pain-related

•Pain duration: 0 to 12 months versus 13 months to 4 years versus 5 or more years

•Baseline pain intensity: CPG intensity score 0 to 3 (low) versus 4 to 7 (medium) versus 8 to 10 (high)

•Baseline pain-related disability: CPG disability score 0 to 3 (low) versus 4 to 7 (medium) versus 8 to 10 (high)

•Baseline depression: HADS depression score <11 versus ≥11

### Analysis of secondary outcomes

#### CPG disability at 6 months

This outcome will be analysed using the same methods as CPG disability at 12 months.

#### CPG pain intensity, HADS Anxiety, HADS Depression, and HEIQ at 6 and 12 months

These outcomes will be analysed using the same methods as CPG disability at 6 and 12 months.

#### PSEQ at 6 and 12 months

These outcomes will be analysed using the same methods as CPG disability at 6 and 12 months, except the individual components of the PSEQ score at 12 weeks will also be included in the imputation model (in addition to the PSEQ score at baseline, 6, and 12 months).

#### CPAQ at 6 and 12 months

These outcomes will be analysed using the same methods as CPG disability at 6 and 12 months, with the exception of how CPAQ at baseline is included in the MI model. We will include the individual questions for CPAQ at 6 and 12 months in the imputation model, and include the overall CPAQ score at baseline For participants who are missing CPAQ at baseline, we will use mean imputation [33]. We chose this approach because CPAQ is a composite of 20 questions including each of these questions at each time point in the imputation model would lead to 60 variables being included (20 questions at baseline, 20 at 6 months, and 20 at 12 months), which may cause problems [34]. Including the overall CPAQ score at baseline rather than the 20 individual questions would reduce the number of variables from 60 to 41.

#### EQ-5D at 6 and 12 months

These outcomes will be analysed using the same analysis model as the primary outcome (that is, a mixed-effects linear regression model, with course as a random effect, adjusted for site of recruitment, age, gender, HADS depression score, and EQ-5D at baseline).

All participants who fully complete the EQ-5D score at either 6 or 12 months will be included in the analysis.

MI will be used to account for participants who are missing the outcome at either 6 or 12 months. The MI strategy will be the same as that for the primary and other secondary outcomes, except instead of imputing the individual components of the EQ-5D score, we will impute the whole score.

#### Census global health question at 6 and 12 months

These outcomes will be analysed using a mixed-effects ordered logistic regression model, with 'course’ as a random effect. Site of recruitment, age, gender, HADS depression score, and the outcome at baseline will be included as fixed covariates.

All participants who completed the Census global health question score at either 6 or 12 months will be included in the analysis.

MI will be used to account for participants who are missing the outcome at either 6 or 12 months. The MI strategy will be the same as that for the primary and other secondary outcomes, except we will impute the whole score (as there are no individual components).

#### Total DDDs up to 12 months post-randomisation for psychotropic drugs, drugs for pain, weak opioids, and strong opioids

These outcomes will be analysed using a mixed-effects linear regression model, with 'course’ as a random effect. Restricted maximum likelihood (REML) will be used. The model will include site of recruitment, age, gender, HADS depression score, and Total DDD in 3 months before randomisation at baseline as covariates. All participants who have data on Total DDD up to 12 months post-randomisation will be included in the analysis. In order to include participants with missing covariates in the analysis, mean imputation will be used to account for missing baseline covariates [33].

#### Proportion of participants using weak opioids and strong opioids at 12 months post-randomisation

These outcomes will be analysed using a mixed-effects logistic regression model, with 'course’ as a random effect. The model will include site of recruitment, age, gender, HADS depression score, and weak or strong (depending on outcome) opioid use at baseline (defined as a prescription for weak or strong) opioids in the 12 weeks before randomization) as covariates. All participants who have data on whether they had had a weak/strong opioid prescription at 12 months will be included in the analysis. Mean imputation will be used to account for missing baseline covariates [33].

### Adherence-adjusted analysis

As a secondary analysis, CPG disability, CPG pain intensity, PSEQ, HADS anxiety, HADS depression, CPAQ, HEIQ, and EQ-5D, all at 12 months will be re-analysed to obtain a complier average causal effect of treatment (CACE) [35,36]. This is because while ITT will give an unbiased estimate of the effect of assigning treatment, it may underestimate the effect of actually receiving the treatment. An analysis using CACE will lead to an unbiased estimate of receiving treatment.

We define 'compliers’ as those who attend more than half of the course (that is, those present for at least 12 of the 24 course components). The compliers can only be observed in the intervention group, where an indicator variable will indentify whether the individual complied. The compliers’ class is unobserved in the control group.

The analysis will be performed using the Stata command ivregress, with the option vce (cluster *clustvar*) to account for clustering by course group. Randomisation will be used as an instrumental variable for treatment received. The main analysis will be adjusted for CPG disability score at baseline, site of recruitment, age, gender, and the HADS depression score at baseline. Analyses will be performed using the same multiply imputed datasets as the primary analysis. The assumptions used for this analysis, as well as other adherence-adjusted analyses to be performed, are listed in the online appendix.

## Abbreviations

CACE: complier average causal effect; COPERS: The Coping with Persistent Pain, Effectiveness Research into Self-management Trial; CPAQ: Chronic Pain Acceptance Questionnaire; CPG: Chronic Pain Grade; GIS: Geographical Information Systems; HADS: Hospital Anxiety and Depression Scale; HEIQ: Health Education Impact Questionnaire; ITT: intention-to-treat; MI: multiple imputation; PSEQ: Pain Self-efficacy Questionnaire; REML: restricted maximum likelihood; SD: standard deviation; SES: socioeconomic status; Total DDD: total defined daily doses

## Competing interests

The authors declare that they have no competing interests.

## Authors’ contributions

BCK, KDO, KH, DC, MU, SJCT, and SE drafted and finalised the statistical analysis plan. SB and SE wrote the statistical analysis sections in the trial protocol. SJCT was the chief investigator for this trial; MU and SE contributed to the design. All authors read and approved the manuscript for publication.

## Supplementary Material

Additional file 1: Methods used to calculate derived variables, and more information on how adherence-adjusted analyses will be performed. Click here for file

## References

[B1] Carnes D Taylor SJ Homer K Eldridge S Bremner S Pincus T Rahman A Underwood M Effectiveness and cost-effectiveness of a novel, group self-management course for adults with chronic musculoskeletal pain: study protocol for a multicentre, randomised controlled trial (COPERS) BMJ Open 2013 3 e002492 23358564 10.1136/bmjopen-2012-002492 PMC3563130

[B2] Carnes D Homer K Underwood M Pincus T Rahman A Taylor SJ Pain management for chronic musculoskeletal conditions: the development of an evidence-based and theory-informed pain self-management course BMJ Open 2013 3 e003534 10.1136/bmjopen-2013-003534 24231458 PMC3831098

[B3] Von Korff M Ormel J Keefe FJ Dworkin SF Grading the severity of chronic pain Pain 1992 50 133 149 10.1016/0304-3959(92)90154-4 1408309

[B4] Nicholas MK The pain self-efficacy questionnaire: taking pain into account Eur J Pain 2007 11 153 163 10.1016/j.ejpain.2005.12.008 16446108

[B5] Zigmond AS Snaith RP The hospital anxiety and depression scale Acta Psychiatr Scand 1983 67 361 370 10.1111/j.1600-0447.1983.tb09716.x 6880820

[B6] McCracken LM Vowles KE Eccleston C Acceptance of chronic pain: component analysis and a revised assessment method Pain 2004 107 159 166 10.1016/j.pain.2003.10.012 14715402

[B7] Osborne RH Elsworth GR Whitfield K The Health Education Impact Questionnaire (heiQ): an outcomes and evaluation measure for patient education and self-management interventions for people with chronic conditions Patient Educ Couns 2007 66 192 201 10.1016/j.pec.2006.12.002 17320338

[B8] Group E The EuroQol instrumnet: EQ-5D 1990 http://www.euroqol.org/eq-5d

[B9] Statistics OfN Census 2011 2011 http://www.ons.gov.uk/census/2011-census

[B10] White IR Horton NJ Carpenter J Pocock SJ Strategy for intention to treat analysis in randomised trials with missing outcome data BMJ 2011 342 d40 10.1136/bmj.d40 21300711 PMC3230114

[B11] Kahan BC Morris TP Assessing potential sources of clustering in individually randomised trials BMC Med Res Methodol 2013 13 58 10.1186/1471-2288-13-58 23590245 PMC3643875

[B12] Andridge RR Shoben AB Muller KE Murray DM Analytic methods for individually randomized group treatment trials and group-randomized trials when subjects belong to multiple groups Stat Med 2014 doi: 10.1002/sim.6083 10.1002/sim.6083 PMC4013262 24399701

[B13] Pals SL Murray DM Alfano CM Shadish WR Hannan PJ Baker WL Individually randomized group treatment trials: a critical appraisal of frequently used design and analytic approaches Am J Public Health 2008 98 1418 1424 10.2105/AJPH.2007.127027 18556603 PMC2446464

[B14] Kahan BC Morris TP Reporting and analysis of trials using stratified randomisation in leading medical journals: review and reanalysis BMJ 2012 345 e5840 10.1136/bmj.e5840 22983531 PMC3444136

[B15] Kahan BC Morris TP Improper analysis of trials randomised using stratified blocks or minimisation Stat Med 2012 31 328 340 10.1002/sim.4431 22139891

[B16] Kahan BC Morris TP Analysis of multicentre trials with continuous outcomes: when and how should we account for centre effects? Stat Med 2013 32 1136 1149 10.1002/sim.5667 23112128

[B17] Kahan BC Accounting for centre-effects in multicentre trials with a binary outcome - when, why, and how? BMC Med Res Methodol 2014 14 20 10.1186/1471-2288-14-20 24512175 PMC3923100

[B18] Hernandez AV Steyerberg EW Habbema JD Covariate adjustment in randomized controlled trials with dichotomous outcomes increases statistical power and reduces sample size requirements J Clin Epidemiol 2004 57 454 460 10.1016/j.jclinepi.2003.09.014 15196615

[B19] Pocock SJ Assmann SE Enos LE Kasten LE Subgroup analysis, covariate adjustment and baseline comparisons in clinical trial reporting: current practice and problems Stat Med 2002 21 2917 2930 10.1002/sim.1296 12325108

[B20] Turner EL Perel P Clayton T Edwards P Hernandez AV Roberts I Covariate adjustment increased power in randomized controlled trials: an example in traumatic brain injury J Clin Epidemiol 2012 65 474 481 10.1016/j.jclinepi.2011.08.012 22169080 PMC3589911

[B21] Kahan BC Morris TP Adjusting for multiple prognostic factors in the analysis of randomised trials BMC Med Res Methodol 2013 13 99 10.1186/1471-2288-13-99 23898993 PMC3733981

[B22] Royston P Altman DG Sauerbrei W Dichotomizing continuous predictors in multiple regression: a bad idea Stat Med 2006 25 127 141 10.1002/sim.2331 16217841

[B23] Campbell H Dean CB The consequences of proportional hazards based model selection Stat Med 2013 10.1002/sim.6083 10.1002/sim.6021 24136328

[B24] Freeman PR The performance of the two-stage analysis of two-treatment, two-period crossover trials Stat Med 1989 8 1421 1432 10.1002/sim.4780081202 2616932

[B25] Kahan BC Bias in randomised factorial trials Stat Med 2013 32 4540 4549 10.1002/sim.5869 23733397

[B26] Raab GM Day S Sales J How to select covariates to include in the analysis of a clinical trial Control Clin Trials 2000 21 330 342 10.1016/S0197-2456(00)00061-1 10913808

[B27] Shuster JJ Diagnostics for assumptions in moderate to large simple clinical trials: do they really help? Stat Med 2005 24 2431 2438 10.1002/sim.2175 15977289

[B28] Rubin DB Multiple Imputation for Nonresponse in Surveys 1987 New York: John Wiley and Sons

[B29] White IR Royston P Wood AM Multiple imputation using chained equations: Issues and guidance for practice Stat Med 2011 30 377 399 10.1002/sim.4067 21225900

[B30] Carpenter JR Goldstein H Kenward MG REALCOM-IMPUTE software for multilevel multiple imputation with mixed response types J Stat Softw 2011 45 5 [epub]

[B31] Andridge RR Quantifying the impact of fixed effects modeling of clusters in multiple imputation for cluster randomized trials Biom J 2011 53 57 74 10.1002/bimj.201000140 21259309 PMC3124925

[B32] Morris TP Kahan BC White IR Choosing sensitivity analyses for randomised trials: principles BMC Med Res Methodol 2014 14 11 10.1186/1471-2288-14-11 24456267 PMC3904008

[B33] White IR Thompson SG Adjusting for partially missing baseline measurements in randomized trials Stat Med 2005 24 993 1007 10.1002/sim.1981 15570623

[B34] Hardt J Herke M Leonhart R Auxiliary variables in multiple imputation in regression with missing X: a warning against including too many in small sample research BMC Med Res Methodol 2012 12 184 10.1186/1471-2288-12-184 23216665 PMC3538666

[B35] Emsley R Dunn G White IR Mediation and moderation of treatment effects in randomised controlled trials of complex interventions Stat Methods Med Res 2010 19 237 270 10.1177/0962280209105014 19608601

[B36] Sussman JB Hayward RA An IV for the RCT: using instrumental variables to adjust for treatment contamination in randomised controlled trials BMJ 2010 340 c2073 10.1136/bmj.c2073 20442226 PMC3230230

