# Supplementary material for: Coping with Persistent Pain, Effectiveness Research into Self-management (COPERS): statistical analysis plan for a randomised controlled trial
Source: Trials. 2014 Feb 15;15:59. doi: 10.1186/1745-6215-15-59 (PMC3930300; doi:10.1186/1745-6215-15-59)
Supplement: Additional file 1: — Methods used to calculate derived variables, and more information on how adherence-adjusted analyses will be performed. [file 1745-6215-15-59-S1.docx]

**Online appendix:**

**The Coping with Persistent Pain, Effectiveness Research into Self-Management (COPERS) trial – statistical analysis plan**

## *Methods of calculating derived variables*

**CPG disability at 6 months**

This is derived in the same method as the CPG disability score at 12 months (as described in the main article).

**CPG pain intensity score at 6 and 12 months.**

This is a composite of three questions which assess the participant’s pain intensity at present, and the maximum and average intensity over the past 6 months. Each question is scored on a scale of 0-10. The outcome is the mean of the three questions, multiplied by 10. Its range is from 0-100, with higher scores indicating worse pain.

**PSEQ (Pain Self-Efficacy Questionnaire) score at 6 and 12 months**

This is a composite of 10 questions which ascertain the participant’s level of confidence to live a normal life despite their pain. Each question is scored on a scale of 0-6. The outcome is the sum of all 10 questions. Its range is 0-60, with higher scores indicating higher levels of confidence.

**HADS (Hospital Anxiety and Depression Scale) Anxiety score at 6 and 12 months**

This is a composite of 7 questions which ascertains the extent of the participant’s anxiety (these are the odd number questions of the HADS questionnaire). Each question has four answers ranging from not experiencing a symptom at all scored as 0, to experiencing a symptom nearly all the time scored as 3. The outcome is the sum of each question. Its range is 0-21, with higher scores indicating more severe anxiety.

**HADS (Hospital Anxiety and Depression Scale) Depression score at 6 and 12 months**

This is a composite of 7 questions which ascertains the extent of the participant’s depression (these are the even number questions of the HADS questionnaire). Each question has four answers ranging from not experiencing a symptom at all scored as 0, to experiencing a symptom nearly all the time scored as 3. The outcome is the sum of each question. Its range is 0-21, with higher scores indicating more severe depression.

**CPAQ (Coping Pain and Acceptance Questionnaire) score at 6 and 12 months**

This is a composite of 20 questions which ascertain the participant’s ability to cope with their pain. Each question is scored on a scale of 0-6, with 0 indicating the statement is never true, and 6 indicating the statement is always true. There are two subscales: Pain Willingness and Activities Engagement. The statements in the Pain Willingness subscale are reverse scored, so that an answer of ‘Always true’ gives a score of 0, and a score of ‘Never true’ gives a score of 6. The outcome is the sum of each question. Its range is 0-120, with higher scores indicating a better ability to cope.

**HEIQ (Health Education Impact Questionnaire) score at 6 and 12 months**

This is a composite of 5 questions which ascertain the extent to which the participant is able to enjoy life. Each question has four answers ranging from Strongly Agree (scored as 4) to Strongly Disagree (scored as 1). The outcome is the sum of each question. It’s range is 4-20, with higher scores indicating more enjoyment in life.

**EQ-5D at 6 and 12 months**

This is a composite of 5 questions which ascertain whether the participant has any problems with mobility, self-care, performing their usual activities, pain or discomfort, or anxiety or depression. Each question has three answers ranging from ‘No problems’ (scored as 1) to the worst category (scored as 3). The outcome score will be derived using the method described in the SPSS manual.

## Drugs Data Analysis

**Total Defined Daily Doses (Total DDD) consumed**

The Total DDD for each drug is defined as:

Total DDD_DrugA_ = (Strength_MedA_ x quantity_MedA_)/DDD_MedA_

The Total DDD for a group of medications (e.g. the Total DDD for opioids) is the sum of the Total DDD for each drug within that medication group (e.g. each drug which is considered an opioid). For example, if there are three drugs (drugs A, B, and C), the TotalDDD_opioid_ is defined as:

TotalDDD_opioid_ = TotalDDD_DrugA_ + TotalDDD_DrugB_ + TotalDDD_DrugC_

The DDD (used in the denominator of the calculation for the TotalDDD) is determined in the first instance by the WHO register, then by precedent in other trials (OPERA and TOIB), and then by clinician consensus. For compound drugs, e.g. co-codamol we will separate out components (paracetamol & codeine) and work out the DDD for each component drug.

**Example**

Consider a participant who has been prescribed 60 tablets of Morphine Sulphate 10 mg during the follow-up period. The Total DDD for this drug can be calculated using the formula:

Total DDD_Morphine_Sulphate_ = (Strength_Morphine_Sulphate_ x quantity_Morphine_Sulphate_)/DDD_Morphine_Sulphate_

Step 1: Calculate DDD_Morphine_Sulphate_ using information provided at <http://www.whocc.no/atc_ddd_index/>. Based on this website, DDD_Morphine_Sulphate_ = 0.1g.

Step 2: Calculate Strength_Morphine_Sulphate_ (the strength of the drug prescribed). This needs to be calculated in the same units as the WHO DDD in step 1 (i.e. it must be converted to grams). Therefore, Strength_Morphine_Sulphate_ = 0.01g (as 10mg = 0.01g).

Step 3: Calculate quantity_Morphine_Sulphate_(quantity prescribed). This is 60 (as 60 tablets were prescribed).

Step 4: Calculate Total DDD_Morphine_Sulphate_ based on information provided from steps 1-3. This is

Total DDD_Morphine_Sulphate_ = (60 *0.01g)/0.1g = 6

**Data**

Medications used over a 15 month period have been collected from GP participant records. We extracted drug name and strength used, plus quantity and the dates i.e. number of times the medication was prescribed. We have used the prescription cost analysis database to attach a cost to each individual preparation used. Using the World Health Organization (WHO)-defined daily dose for each drug we will generate number of days of medication used by *British National Formulary* chapter and subchapter.

### Outcomes

We consider the following outcomes:

1. Total Defined Daily Doses (Total DDD) consumed of psychotropic drugs (Table 1) up to 12 months post randomisation
2. Total DDD consumed of all analgesics up to 12 months post randomisation
3. Total DDD consumed of weak opioids up to 12 months post-randomisation (as defined by BNF 4.7.2 are codeine, dihydrocodeine and meptazionol )
4. Total DDD consumed of all NSAID analgesics (oral and topical combined) up to 12 months post randomisation
5. Total DDD consumed of all CNS drugs for neuropathic pain (see Table 1) up to 12 months post-randomisation
6. Total DDD consumed of strong opioids up to 12 months post-randomisation (as defined by BNF 4.7.2, all opioids prescribed other than the ones listed above as weak)

Calculations for psychotropic drugs will be based on BNF subchapters 4.1, and 4.3, opioids based on BNF paragraph 4.7.2, and analgesics including opioids based on BNF paragraphs 4.7.1, 4.7.2, 4.7.3, and paragraphs10.1.1, 10.2.2, and 10.3.2.

### Relevant Drugs

We will work out DDD for BNF chapter 4 and 10 groups of drugs, these are drugs used for treating chronic pain (see table below). We will exclude all drugs administered as injections, but we will include soluble drugs, gels and liquids.

## *Adherence-adjusted analysis*

As a secondary analysis, CPG disability, CPG pain intensity, PSEQ, HADS anxiety, HADS depression, CPAQ, HEIQ, and EQ-5D, all at 12 months will be re-analysed to obtain a complier average causal effect of treatment (CACE). We define ‘compliers’ as those who attend more than half of the course (i.e. those present for at least 12 of the 24 course components). The compliers can only be observed in the intervention arm, where an indicator variable will indentify whether the individual complied. The compliers’ class is unobserved in the control arm.

We assume the Stable Unit Treatment Value Assumption (SUTVA), namely: (a) no interference between study units (the outcome for each participant depends only on their own treatment assignment and not the treatment assignment of any other participant), and (b) consistency, which implies that the observed outcome for each participant will equal one of the potential outcomes.

In addition for identification, we assume (a) monotonicity: there are no defiers; and (b) exclusion restriction: treatment allocation only has an effect on outcome through treatment received and the effect of assignment is completely mediated by treatment exposure. .

Under the assumptions stated above, we will use randomisation as an instrumental variable for treatment received and obtain a CACE treatment estimate by a two-stage least squares instrumental variable regression (using STATA command ivregress). We will run two analyses, one without any covariates and another one which includes all the baseline covariates included in the primary analysis models, namely CPG disability score at baseline, site of recruitment, age, gender, and the HADS depression score at baseline, and assumes a structural mean model. The covariate-adjusted CACE will be considered the primary CACE analysis.

We will assume that missing data are missing at random and use the same multiply imputed datasets produced for the primary analyses. We will analyse each of multiply imputed sets, using robust estimation for the variance (using the option vce(cluster *clustvar*)) to account for the possible clustering by course group; finally obtaining MI estimates using Rubin’s rules as before.

**Table 1- Pain related drugs**

|  | **Chapter** | **Subchapter** | **Paragraph** | **Comments** |
| --- | --- | --- | --- | --- |
| Psychotropic drugs | 4. Central Nervous System | 4.1. Hypnotics and Anxiolytics | 4.1.1 Hypnotics  4.1.2.Anxiolytics | NOT: chloral and derivatives, clomethiazole or antihistamines |
|  |  | 4.3. Antidepressant drugs | 4.3.2 Monoamine-oxidase inhibitors  4.3.3. Selective serotonin re-uptake inhibitors  4.3.4 Other anti depressant drugs |  |
| Analgesic drugs |  | 4.7 Analgesics | 4.7.1 Non opioid analgesics  4.7.2. Opioid analgesics  4.7.3 Neuropathic and functional pain | 4.8.1 Gabapentin and pregabalin feature as an anti-epileptic but also feature in 4.7.3 Neuropathic and functional pain  For this analysis 4.3.1 tricyclic anti-depressants are included in section 4.7.3 |
|  | 10. Musculoskeletal and joint diseases (exclude steroids, DMARDS) | 10.1 Drugs used in rheumatic diseases and gout | 10.1.1 Non-steroidal anti inflammatories | Exclude aspirin  No steroids |
|  |  | 10.2 Drugs used in neuromuscular disorders | 10.2.2 Skeletal muscle relaxants |  |
|  |  | 10.3 Drugs for the relief of soft tissue inflammation | 10.3.2 Rubefacients and other topical anti-rheumatics | Not enzymes |
